# Biographical

**Published:** 1897-06

**Authors:** 


### Biographical.

Dr. J. A. Robinson, formerly a resident of Jackson, Mich., died at the home of his son, Dr. A. Robinson, in Grand Rapids, on March 3d, in the eighthy-fifth year of his age. His strength had been gradually failing for two years; about a week before his death he was attacked with a severe influenza, which was the immediate cause of his death. He was born in Concord, Mass. May 31st, 1812. He was of Puritan stock; a desendant of Rev. John Robinson of Leyden, who expected to sail with the pilgrims in the Mayflower, but was prevented by sickness. His grandfather was a lieutenant in the revolutionary army; his father, Wm. Robinson, was a prominent citizen of Concord, Mass., and his mother, whose maiden name was Martha Cogswell, was also of New England birth. He married Harriet Emelia Brown in 1832, the family consisted of nine children.

In 1835 he began the practice of dentistry with Dr. George Mansfield of Lowell, Mass., and in 1837 he entered upon the practice of his profession in Old Salem, of that State, where he remained until 1852 when he removed to Cleveland, Ohio. In 1858 he removed to Jackson, Michigan, where he continued in the practice of his profession until about two years before his death; his wife having passed away about one year before he left Jackson. Dr. Robinson gives a sketch of his early life, and especially so far as it pertains to his study of dentistry and his entrance to the profession; and its attendant embarrassments and difficulties. In the August number of the Dental Register for 1896, on page three hundred and ninety-six is given a sketch of his early history, which need not be repeated here.

Dr. Robinson was naturally an ardent reformer throughout his life. He followed his convictions where ever they might lead him, and he never hesitated to announce them whenever occasion for it occurred. He was ardently opposed to slavery, and was among the first to take a decided stand against that iniquity. He was an ardent temperance advocate, a man of strong religious convictions, and was for many years engaged in Mission Sunday School work. He was a zealous supporter of the government during the civil war, and gave one of his sons, Captain Wm. T. Robinson, of the Fourth Michigan Infantry in the service of his country's cause. He was in a broad sense a patriot and humanitarian. He was for many years a writer for the press, usually upon questions of reform or general public interest. He had also a poetic vein in his make-up to which he gave expression in pleasing and striking verse, but, even these, always had the impress of his natural characteristics. Many of his poems attracted wide attention. Dr. Robinson possessed a warm genial nature, ardently attached to his friends. His aim seemed to be to make everybody about him and within his reach happy.

When he ceased the practice of his profession, about two years ago, he was the oldest practitioner in the State of Michigan, if not in the United States. He made many advanced steps in the practice of his profession that have been greatly serviceable, though the origin of which is not generally known. He was unselfish in a marked degree, caring more for the comfort and welfare of others than for himself. He lived to accomplish his work to the full, and in a manner that will bring joy to his children and a blessing to humanity.

